# Investigation of the safety and protective efficacy of an attenuated and marker *M. bovis*-BoHV-1 combined vaccine in bovines

**DOI:** 10.3389/fimmu.2024.1367253

**Published:** 2024-04-04

**Authors:** Sen Zhang, Guoxing Liu, Yisheng Zhang, Chen Wang, Xiaowen Xu, Yuhao Zhao, Zhijie Xiang, Wenying Wu, Li Yang, Jianguo Chen, Aizhen Guo, Yingyu Chen

**Affiliations:** ^1^State Key Laboratory of Agricultural Microbiology, Hubei Hongshan Laboratory, College of Veterinary Medicine, Huazhong Agricultural University, Wuhan, China; ^2^Hubei International Scientific and Technological Cooperation Base of Veterinary Epidemiology, The Cooperative Innovation Center for Sustainable Pig Production, Wuhan, China; ^3^Key Laboratory of Development of Veterinary Diagnostic Products, Ministry of Agriculture and Rural Affair, Wuhan, China; ^4^Wuhan Keqian Biology Co., Ltd, Research and Development Department, Wuhan, China

**Keywords:** BRD, *M. bovis*, BoHV-1, attenuated and marker vaccine, protective efficacy

## Abstract

Bovine respiratory disease (BRD) is one of the most common diseases in the cattle industry worldwide; it is caused by multiple bacterial or viral coinfections, of which *Mycoplasma bovis* (*M. bovis*) and bovine herpesvirus type 1 (BoHV-1) are the most notable pathogens. Although live vaccines have demonstrated better efficacy against BRD induced by both pathogens, there are no combined live and marker vaccines. Therefore, we developed an attenuated and marker *M. bovis*-BoHV-1 combined vaccine based on the *M. bovis* HB150 and BoHV-1 gG-/tk- strain previously constructed in our lab and evaluated in rabbits. This study aimed to further evaluate its safety and protective efficacy in cattle using different antigen ratios. After immunization, all vaccinated cattle had a normal rectal temperature and mental status without respiratory symptoms. CD4^+^, CD8^+^, and CD19^+^ cells significantly increased in immunized cattle and induced higher humoral and cellular immune responses, and the expression of key cytokines such as IL-4, IL-12, TNF-α, and IFN-γ can be promoted after vaccination. The 1.0 × 10^8^ CFU of *M. bovis* HB150 and 1.0 × 10^6^ TCID_50_ BoHV-1 gG-/tk- combined strain elicited the most antibodies while significantly increasing IgG and cellular immunity after challenge. In conclusion, the *M. bovis* HB150 and BoHV-1 gG-/tk- combined strain was clinically safe and protective in calves; the mix of 1.0 × 10^8^ CFU of *M. bovis* HB150 and 1.0 × 10^6^ TCID_50_ BoHV-1 gG-/tk- strain was most promising due to its low amount of shedding and highest humoral and cellular immune responses compared with others. This study introduces an *M. bovis*-BoHV-1 combined vaccine for application in the cattle industry.

## 1 Introduction

Bovine respiratory disease (BRD) is a widespread disease and one of the greatest challenges in the livestock industry. It is a leading cause of morbidity, mortality, and economic loss in cattle (1), accounting for approximately 70%–80% of total morbidity in feedlots, especially among newborn calves (2, 3). Over 90% of feedlots in the United States report BRD as the most prevalent disease, with an estimated annual cost ranging from USD 1 to 3 billion (4, 5); in Australia, the documented morbidity and mortality rates for BRD were 18% and 2.1%, respectively, with an average net loss of AUD 1647.53 per death (6). Currently, the pathogenesis of BRD is believed to be orchestrated through the synergistic interaction of various bacteria and viruses in addition to changes in the host and environment (7).

Several pathogens can contribute to BRD such as bovine herpesvirus type 1 (BoHV-1), bovine viral diarrhea virus, bovine respiratory syncytial virus, *Mycoplasma bovis* (*M. bovis*), *Mannheimia haemolytica* (*Mh*), and *Pasteurella multocida* (*Pm*) (8). In China, *M. bovis* was first isolated in 2008 from beef cattle with pneumonia (9); the overall prevalence of BoHV-1 among cattle was then found to be approximately 40% (10). In Xinjiang and Inner Mongolia, the nucleic acid positive rate has reached 9% and 24.83%, respectively (10, 11). Some live vaccines developed by Bimeda Biologicals and Boehringer Ingelheim Animal Health target specific pathogens of BRD like *M. bovis and* BoHV-1. However, there are currently no combined live and marker vaccines.

In our previous study, we developed two single-component vaccines named *M. bovis* HB150 and BoHV-1 gG-/tk- that protected bovine immune systems (12, 13). We also showed the efficacy of *M. bovis* HB150 and BoHV-1 gG-/tk- as a combination vaccine in rabbits (14). Here, we evaluated the safety, immunization efficacy, and protection efficacy of the *M. bovis*–BoHV-1 combined vaccine in cattle for the first time. In addition, the combined vaccine can also achieve the effect of preventing multiple pathogens with a single inoculation, highlighting its potential to prevent and control BRD in the cattle industry.

## 2 Materials and methods

### 2.1 Cells and viruses

Wild-type BoHV-1 HB06 (GenBank accession number: AJ004801.1), BoHV-1 gG^-^/tk^-^ strain, *M. bovis* HB0801 (GenBank accession number: CP002058.1) strain and *M. bovis* HB150 strain were maintained in the State Key Laboratory of Agricultural Microbiology. Madin-Darby bovine kidney cells (MDBK) were procured from the China Institute of Veterinary Drug Control.

### 2.2 Culture of *M. bovis* and BoHV-1

*M. bovis* and BoHV-1 were cultured as previously described (15, 16). Briefly, *M. bovis* HB0801 and HB150 strains were cultured in PPLO complete medium at 37°C in a 5% CO_2_ incubator for 40–48h. BoHV-1 strains HB06 and BoHV-1 gG^-^/tk^-^ were cultured in Dulbecco’s modified Eagle’s medium (DMEM) supplemented with 10% fetal bovine serum (Inner Mongolia Opcel Biotechnology Co., Ltd., Hohhot, China) using MDBK cells at 37°C in a 5% CO_2_ incubator.

The initial doses of the *M. bovis* HB150 and BoHV-1 gG^-^/tk^-^ were 1.0 × 10^8^ CFU and 1.0 × 10^6^ TCID_50_, respectively. The ratios of 1:1, 1:2, and 2:1 were prepared as follows: 1.0 × 10^8^ CFU *M. bovis* HB150 with 1.0 × 10^6^ TCID_50_ BoHV-1 gG^-^/tk^-^, 1.0 × 10^8^ CFU *M. bovis* HB150 with 2.0 × 10^6^ TCID_50_ BoHV-1 gG^-^/tk^-^, and 2.0 × 10^8^ CFU *M. bovis* HB150 with 1.0 × 10^6^ TCID_50_ BoHV-1 gG^-^/tk^-^.

### 2.3 Animal experiments

A total of 33 two- to four-month-old Holstein dairy cows purchased from pasture that were seronegative for *M. bovis*, BoHV-1, *Pasteurella*, and *Mannheimia haemolytica* were divided into 11 groups. All cattle were housed in isolation to prevent cross-infection. According to the antigen dose, groups 1 and 2 were immunized with 1.0 × 10^8^ CFU *M. bovis* HB150 mixed with 1.0 × 10^6^ TCID_50_ BoHV-1 gG^-^/tk^-^ strain (presented as 1:1); groups 3 and 4 were immunized with 1.0 × 10^8^ CFU *M. bovis* HB150 mixed with 2.0×10^6^ TCID_50_ BoHV-1 gG^-^/tk^-^ strain (presented as 1:2); groups 5 and 6 were immunized with 2.0 × 10^8^ CFU *M. bovis* HB150 mixed with 1.0 × 10^6^ TCID_50_ BoHV-1 gG^-^/tk^-^ strain (presented as 2:1), respectively; group 7 was immunized with 1.0 × 10^8^ CFU *M. bovis* HB150 strain; group 8 was immunized with 1.0 × 10^6^ TCID_50_ BoHV-1 gG^-^/tk^-^ strain; groups 9 and 10 were inoculated with PPLO complete medium or DMEM, respectively. Group 11 served as a control. **Table 1** summarizes the study design. All experimental groups were then challenged with 1.0 × 10^9^ CFU *M. bovis* HB0801 strain or 4.0 × 10^7^ TCID_50_ BoHV-1 HB06 strain at 28 days after immunization. Nasal swabs were collected daily for 14 days after immunization and challenge, and blood samples were collected weekly until the end of the experiment. Samples were stored at −80°C. Animals were euthanized at the end of the experiment, and lung tissue, spleen tissue and trigeminal nerve samples were collected for follow-up experiments.

**Table 1:** Animal immunization and challenge information.

Group	Vaccination strain and dose*	No	Challenge strain and dose**
1	1.0×10^8^ CFU *M.bovis* HB150 1.0×10^6^ TCID_50_ BoHV-1 gG^-^/tk^-^	3	1.0×10^9^ CFU *M.bovis* HB0801
2	1.0×10^8^ CFU *M.bovis* HB150 1.0×10^6^ TCID_50_ BoHV-1 gG^-^/tk^-^	3	4.0×10^7^ TCID_50_ BoHV-1 HB06
3	1.0×10^8^ CFU *M.bovis* HB150 2.0×10^6^ TCID_50_ BoHV-1 gG^-^/tk^-^	3	1.0×10^9^ CFU *M.bovis* HB0801
4	1.0×10^8^ CFU *M.bovis* HB150 2.0×10^6^ TCID_50_ BoHV-1 gG^-^/tk^-^	3	4.0×10^7^ TCID_50_ BoHV-1 HB06
5	2.0×10^8^ CFU *M.bovis* HB150 1.0×10^6^ TCID_50_ BoHV-1 gG^-^/tk^-^	3	1.0×10^9^ CFU *M.bovis* HB0801
6	2.0×10^8^ CFU *M.bovis* HB150 1.0×10^6^ TCID_50_ BoHV-1 gG^-^/tk^-^	3	4.0×10^7^ TCID_50_ BoHV-1 HB06
7	1.0×10^8^ CFU *M.bovis* HB150	3	1.0×10^9^ CFU *M.bovis* HB0801
8	1.0×10^6^ TCID_50_ BoHV-1 gG^-^/tk^-^	3	4.0×10^7^ TCID_50_ BoHV-1 HB06
9	Complete PPLO medium	3	1.0×10^9^ CFU *M.bovis* HB0801
10	DMEM medium	3	4.0×10^7^ TCID_50_ BoHV-1 HB06
11	Blank Control	3	\

*Dissolve the vaccine in 2 mL of saline solution. All vaccines were dropped to the nasal cavity using a 2 mL syringe, each nasal cavity was inoculated with 1 mL of vaccine.

**M. bovis HB0801 was challenged through tracheal injection, BoHV-1 HB06 was challenged through intranasal inoculation. The detailed description of the intratracheal inoculation procedure are as follows:

One person should lift the head of the cattle while the other locates the trachea in the neck. The trachea will feel hard and threaded. A 2mL syringe was used to slowly inject M. bovis HB0801 into the trachea, when the cattle made a swallowing sound, indicating that the challenge was finished.

### 2.4 Clinical evaluation and sample collection

Clinical signs including rectal temperature and mental and respiratory status were continuously monitored for 14 days post immunization and post challenge. A thermometer was inserted approximately 10 cm into the rectum until the temperature stopped changing. The rectal temperature of the cattle was measured in the morning and afternoon before the cattle were fed; the average value was recorded.

Nasal swabs were collected daily for 14 days after immunization and challenge. Samples were fully vortexed in tubes containing 1 mL sterile PBS, filtered through a 0.45-µm filter, and stored at −20°C for PCR/RT-PCR and *M. bovis* counting. Blood samples were collected weekly for antibody and cytokine detection until the end of the experiment. Serum was prepared as follows: whole blood without anticoagulant was allowed to clot naturally at room temperature, followed by centrifugation at 4°C, 4000 rpm for 10 minutes. The supernatant was collected as serum. Lungs and spleens and trigeminal nerve samples were collected 28 days after challenge. Tissue samples were fixed in 4% paraformaldehyde for 48 h and then embedded in paraffin. Hematoxylin-eosin staining was performed on the sections followed by histopathological examination.

### 2.5 Virus and bacteria shedding

Genomes of nasal swabs were extracted and amplified by PCR/RT-PCR to detect the shedding of *M. bovis* HB150 using the *uvrC* gene and BoHV-1 gG^-^/tk^-^ using glycoprotein G *gG* and thymidine kinase *tk* genes as previously described under the following reaction conditions (12, 16): *M. bovis* uvrC gene PCR reaction conditions: 95°C for 3 min; 95°C for 15 s, 55°C for 15 s, 72°C for 30 s, 35 cycles; 72°C for 5 min; BoHV-1 gG PCR reaction conditions: 95°C for 3 min; 95°C for 15 s, 60.5°C for 15 s, 72°C for 40 s, 35 cycles; 72°C for 5 min; BoHV-1 tk PCR reaction conditions: 95°C for 3 min; 95°C for 15 s, 60.5°C for 15 s, 72°C for 30 s, 35 cycles; 72°C for 5 min.

To quantify the shedding of *M. bovis* HB0801 after challenge, the treated nasal swabs were serially diluted 10-fold; 100 μl of the diluted filtrate were then inoculated into PPLO solid medium and incubated at 37°C in a 5% CO_2_ incubator for 3–5 days. When typical “fried eggs shaped” colonies grew on the solid medium, the original concentration was calculated based on the dilution factor.

BoHV-1 HB06 shedding after challenge was detected from the DNA extracted from nasal swabs used for RT-PCR using the envelope glycoprotein B *gB* gene. Trigeminal nerve samples were minced, and genomes were extracted for RT-PCR detection. The program for BoHV-1 gB gene was as follows: 95°C for 30 s; 95°C for 10 s, 60°C for 20 s, 40 cycles; 95°C for 15s, 60°C for 20 s, 95°C for 15s.

### 2.6 Serum antibody of *M. bovis*

Serum antibodies against *M. bovis* were identified by competitive ELISA as previously described (14). In short, the fourfold-diluted test serum as well as positive and negative serum controls with HRP-labeled monoclonal antibodies were added to the *M. bovis* p579 protein-coated plate and incubated at 37°C for 60 min. After washing, 100 µl of substrate chromogenic solution were added and incubated at room temperature (22 °C-25°C) away from light for 10 min; the OD_450nm_ value was read immediately after stopping the reaction. The blocking rate (PI value) was calculated as follows:

Blocking rate = (1-S/N)×100%, S = sample OD_450nm_; N = mean OD_450nm_ of negative control serum. Conditions for the establishment of the test: 0.65 < OD_450nm_ negative control < 2.0, PI_positive control_ > 60%. PI_sample_ ≥ 41% means positive; PI_sample_ < 41% means negative.

### 2.7 Neutralization assay

Inactivated serum (56°C for 30 min) was serially diluted in a 96-well cell culture plate and then incubated with 100 TCID_50_ BoHV-1 HB06 virus at 37°C in a 5% CO_2_ incubator for 1 h. The serum-virus mixture was then transferred to a 96-well cell culture plate containing MDBK cells and cultured in a 5% incubator at 37°C for three days. Neutralizing antibody titers, the highest serum dilutions that inhibit BoHV-1 infection, were calculated using the Reed-Muench method.

### 2.8 Detection of cytokines and ELISA antibodies

Commercial ELISA kits (Jiangsu Meimian Industrial Co., Ltd., Yancheng, China) were used to detect changes in serum cytokine and antibody levels using IL-4, IL-10, IL-12, TNF-α, IFN-γ, sIgA, IgG, and BoHV-1 gB antibodies.

### 2.9 Multicolor fluorescence *in situ* hybridization

Fixed lung tissues were embedded in paraffin, sectioned, and dewaxed. The sections were digested with proteinase K and washed three times with PBS. Subsequently, pre-hybridization, hybridization and washing after hybridization were performed sequentially. The slices were incubated with anti-CD11b, CD11c, CD19, CD4, and CD8 primary antibodies (Servicebio Biotechnology Co., Ltd., Wuhan, China) followed by the corresponding signal probe for hybridization at 42°C for 3h. Then pre-hybridization, hybridization and washing after hybridization were applied again. The corresponding signal probe was then added and incubated at 40°C for 45 min. After this, adding the hybridization solution containing the fluorescently labeled signal probe and incubating at 42°C for 3h. Finally, nuclei were stained with DAPI.

### 2.10 Ethics statement

The animal experiment was approved by the Animal Experiment Ethics Committee of Huazhong Agricultural University and conducted in strict accordance with the Guidelines for the Care and Use of Laboratory Animals of Wuhan, Hubei, China (Huazhong Agricultural University Ethics Approval Number: HZAUCA-2023-0038).

### 2.11 Statistical analysis

Shapiro-Wilk normality test was used for normal distribution. One-way ANOVA was subsequently used to detect significant differences between groups, where *p* < 0.05 (*), *p* < 0.01 (**), *p* < 0.001 (***), or *p* < 0.0001 (****) were considered to be significant statistical differences. Error bars indicate the standard error of the mean.

## 3 Results

### 3.1 Clinical signs

**Figure 1:**
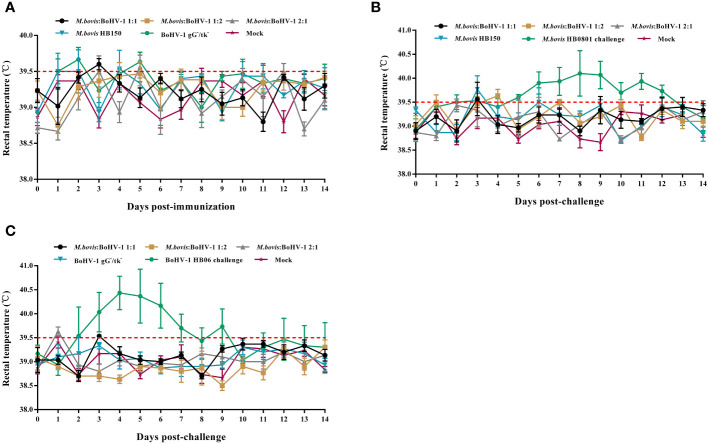
Rectal temperature changes after vaccination **(A)** and *M. bovis* HB0801 **(B)** challenge and BoHV-1 HB06 **(C)** challenge, respectively.

All animals in the immunized groups had normal rectal temperature and mental status without obvious respiratory signs during the entire observation period (**Figure 1A**). The unimmunized groups of cattle experienced rectal temperatures surpassing 39.5°C between days 5 and 12 following the challenge with *M. bovis*, and between days 2 and 9 after challenge with BoHV-1. (**Figures 1B, C**). All cattle in the *M. bovis* HB0801 challenge group showed obvious clinical signs such as decreased feed intake, coughing, profuse salivation accompanied by lacrimation and increasing respiratory rate. Two cattle in the BoHV-1 HB06 challenge group presented with nasal secretion, ocular secretions, coughing and typically nasal mucosal bleeding. These results confirmed that cattle were successfully challenged with *M. bovis* HB0801 and BoHV-1 HB06 and that the developed vaccine was safe.

### 3.2 Detection of *M. bovis* and BoHV-1 shedding

#### 3.2.1 *M. bovis* HB150 and BoHV-1 gG^-^/tk^-^ shedding after immunization

Groups immunized with antigen ratios of 1:1 and 1:2 stopped shedding on the 11th day after immunization, whereas 2:1 and *M. bovis* HB150 groups stopped shedding on the 12th and 13th days, respectively. Over 50% of animals in the 1:1, 1:2, and 2:1 groups continued shedding on day 9 post immunization, whereas the condition lasted one more day in the *M. bovis* HB150 immunization group (**Table 2**).

**Table 2:** Shedding of *M. bovis* HB150 and BoHV-1 gG-/tk- after immunization.

Group		Days post immunization (%)													
		1	2	3	4	5	6	7	8	9	10	11	12	13	14
1 and 2 (1:1)	*M. bovis*	6/6 (100)	6/6 (100)	6/6 (100)	6/6 (100)	6/6 (100)	5/6 (83.3)	3/6 (50)	3/6 (50)	3/6 (50)	1/6 (16.7)	0/6 (0)	0/6 (0)	0/6 (0)	0/6 (0)
	BoHV-1	6/6 (100)	6/6 (100)	6/6 (100)	6/6 (100)	6/6 (100)	6/6 (100)	6/6 (100)	2/6 (33.3)	2/6 (33.3)	1/6 (16.7)	1/6 (16.7)	1/6 (16.7)	0/6 (0)	0/6 (0)
3 and 4 (1:2)	*M. bovis*	6/6 (100)	6/6 (100)	6/6 (100)	6/6 (100)	6/6 (100)	5/6 (83.3)	4/6 (66.7)	4/6 (66.7)	3/6 (50)	2/6 (33.3)	0/6 (0)	0/6 (0)	0/6 (0)	0/6 (0)
	BoHV-1	6/6 (100)	6/6 (100)	6/6 (100)	6/6 (100)	6/6 (100)	6/6 (100)	6/6 (100)	3/6 (50)	3/6 (50)	2/6 (33.3)	2/6 (33.3)	1/6 (16.7)	1/6 (16.7)	0/6 (0)
5 and 6 (2:1)	*M. bovis*	6/6 (100)	6/6 (100)	6/6 (100)	6/6 (100)	6/6 (100)	6/6 (100)	4/6 (66.7)	4/6 (66.7)	4/6 (66.7)	2/6 (33.3)	1/6 (16.7)	0/6 (0)	0/6 (0)	0/6 (0)
	BoHV-1	6/6 (100)	6/6 (100)	6/6 (100)	6/6 (100)	6/6 (100)	6/6 (100)	6/6 (100)	2/6 (33.3)	2/6 (33.3)	1/6 (16.7)	1/6 (16.7)	0/6 (0)	0/6 (0)	0/6 (0)
7 (*M. bovis* HB150)		3/3 (100)	3/3 (100)	3/3 (100)	3/3 (100)	3/3 (100)	3/3 (100)	3/3 (100)	3/3 (100)	2/3 (66.7)	2/3 (66.7)	0/3 (0)	1/3 (33.3)	0/3 (0)	0/3 (0)
8 (BoHV-1 gG-/tk-)		3/3 (100)	3/3 (100)	3/3 (100)	3/3 (100)	3/3 (100)	3/3 (100)	3/3 (100)	1/3 (33.3)	2/3 (66.7)	1/3 (33.3)	1/3 (33.3)	1/3 (33.3)	0/3 (0)	0/3 (0)
11 (Mock)		0/3 (0)	0/3 (0)	0/3 (0)	0/3 (0)	0/3 (0)	0/3 (0)	0/3 (0)	0/3 (0)	0/3 (0)	0/3 (0)	0/3 (0)	0/3 (0)	0/3 (0)	0/3 (0)

No shedding was detected in 1:1 and BoHV-1 gG^-^/tk^-^ groups on day 13 post immunization, and the 1:2 and 2:1 groups stopped shedding 14 days and 12 days post immunization, respectively. The control group showed no viral shedding throughout the observation period. On day 7 post immunization, more than 50% of animals in the 1:1 and 2:1 and BoHV-1 gG^-^/tk^-^ groups continued to shed, and 50% of the animals in the 1:2 group were shedding by day 9 post immunization (**Table 2**). Thus, the 1:2 mixed immunization group showed the highest and most persistent levels of shedding of both pathogens among all mixed immunization groups, followed by the 1:1 and 2:1 mixed immunization groups.

#### 3.2.2 *M. bovis* HB0801 and BoHV-1 HB06 shedding after challenge

**Figure 2:**
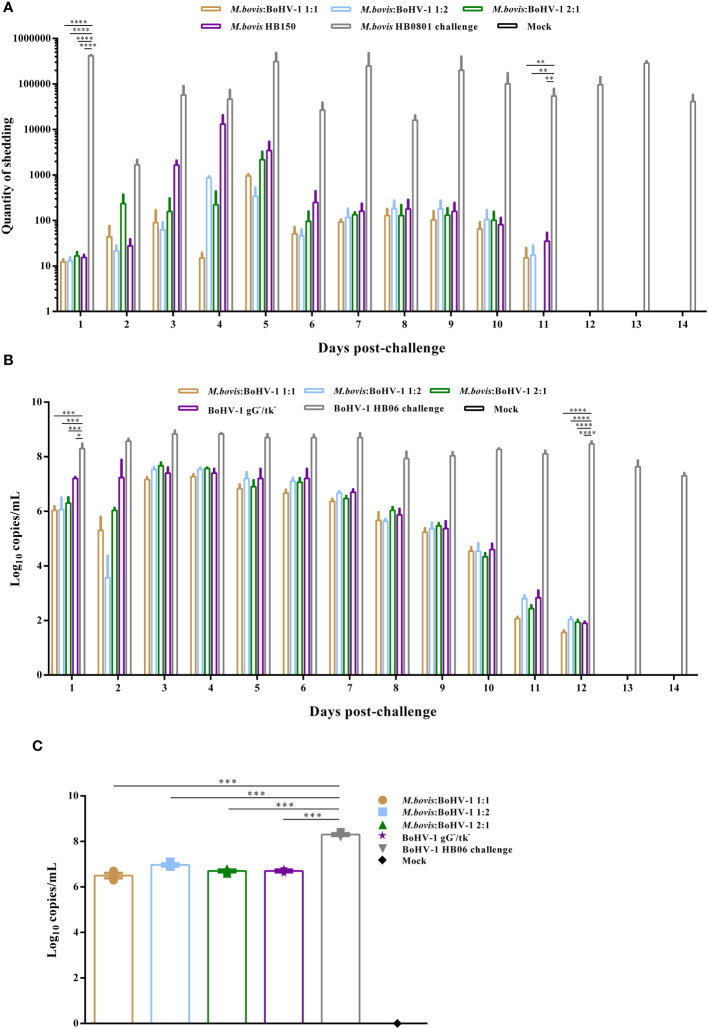
Detection of *M. bovis* HB0801 and BoHV-1 HB06 shedding after challenge. Nasal swabs of calves immunized with different antigenic ratios were collected every day. **(A)** Nasal swabs from groups challenged with *M. bovis* HB0801. **(B)** Nasal swabs from groups challenged with BoHV-1 HB06. **(C)** Trigeminal nerve samples from groups challenged with BoHV-1 HB06.

*M. bovis* HB150 and 2:1 groups continued to shed *M. bovis* HB0801 after challenge, peaking on days 4 and 5, respectively. By day 12, *M. bovis* HB0801 was not detected in any of the vaccinated groups. The non-immune but challenged group shed a lot of *M. bovis* HB0801 during the observation period, which peaked at 10^5^/mL and significantly differed from other vaccinated groups (*p* < 0.01) (**Figure 2A**).

BoHV-1 HB06 shedding after challenge was measured using RT-PCR. High titers of BoHV-1 HB06 were detected in all immunized groups during the first 10 days and peaked on days 3 and 4 after challenge, where the 2:1 group reached 10^7.7^/mL but decreased by day 11 and vanished by day 13. However, no significant difference was found between immunized groups during the entire observation period. The non-immune challenge group continued shedding at high levels after challenge, peaking at 10^8.8^/mL on day 4 after challenge; there was a significant difference between it and immunized groups until day 13 (*p* < 0.0001) (**Figure 2B**).

We also detected shedding of BoHV-1 HB06 in the trigeminal nerve at the end of the experiment. All immunized groups showed a significant decrease in the shedding of BoHV-1 HB06 for about two titers compared to the nonimmune challenge group (*p* < 0.001), but there was no difference between immunized groups (**Figure 2C**). Ultimately, the 1:1 group shed the least compared to the other mixed immunization groups after challenge with *M. bovis* HB0801 or BoHV-1 HB06.

### 3.3 Antibody response

#### 3.3.1 *M. bovis* serum antibody levels

**Figure 3:**
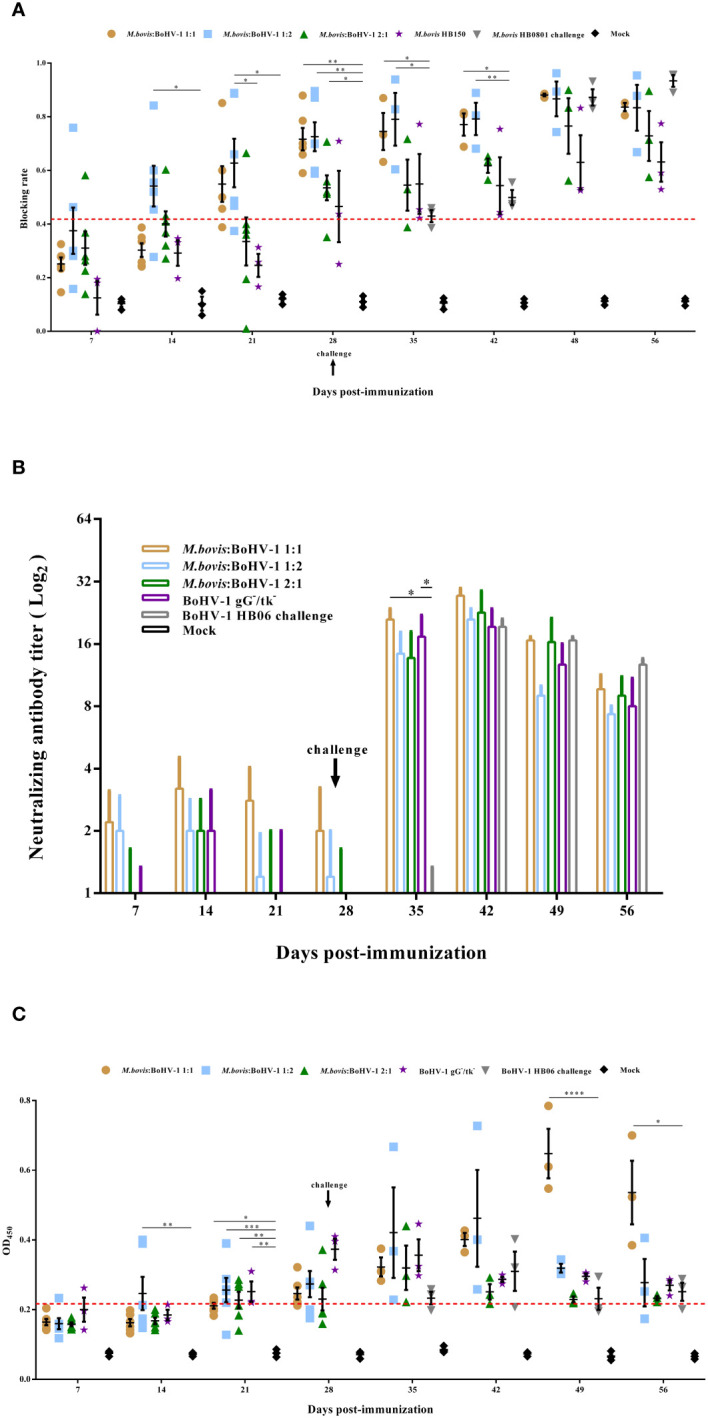
Humoral immune responses induced by *M. bovis*-BoHV-1 bivalent vaccine post- immunization and challenge. Serum was collected weekly to determine **(A)** *M. bovis* specific serum antibody and **(B)** BoHV-1 neutralizing antibody titers and **(C)** BoHV-1 gB antibody.

Competitive ELISA was used to measure the level of *M. bovis* serum antibodies. The antibody level of the 1:2 group was significantly higher than that of the control from days 14 to 28 after immunization (*p* < 0.05), and the blocking rate reached 0.725 at 28 days after immunization. At the same time, the blocking rate of the 1:1 and 2:1 groups was significantly higher than that of the control 28 days post immunization (*p* < 0.05). From day 7 to 14 post challenge, 1:1 and 1:2 groups displayed significantly higher antibody titer compared to the non-immunized groups (*p* < 0.05) (**Figure 3A**).

#### 3.3.2 BoHV-1-neutralizing antibody response

A serum neutralization assay was used to measure the levels of BoHV-1-neutralizing antibodies in cattle. All immunized groups showed low levels of neutralization titer until 7 days post challenge; at this time, 1:1 and BoHV-1 gG^-^/tk^-^ groups induced significantly higher neutralization titer compared to the nonimmunized but challenged group (*p* < 0.05). At 14 days after challenge, the 1:1 group induced the highest levels of neutralizing antibody titer in all experimental groups during the entire observation period, which reached 1:27.3. Calves in the control group did not produce any neutralizing antibodies throughout the experimental period (**Figure 3B**).

#### 3.3.3 BoHV-1 serum-specific gB antibody

gB antibody levels increased slowly in all cattle after immunization, but the immunized groups still had significantly higher antibody levels compared with the control on day 21 post immunization (*p* < 0.05). Notably, all cattle in the 1:1 and BoHV-1 gG^-^/tk^-^ groups turned positive 28 days after immunization, whereas two and four cattle in the 1:2 and 2:1 groups, respectively, remained negative. After BoHV-1 HB06 challenge, gB antibody levels spiked in all immunized groups, particularly in the 1:1 group, whose levels were significantly higher than the non-immune but challenged group from days 21 to 28 post challenge (*p* < 0.05), and peaked on day 21 to 0.647 (OD_450nm_ > 0.215 was considered as antibody positive) on average (**Figure 3C**). Thus, the 1:1 mixed immunization group induced the highest and most persistent *M. bovis* and BoHV-1 antibody titers among all immunization groups.

### 3.4 IgA and IgG titers in calves

**Figure 4:**
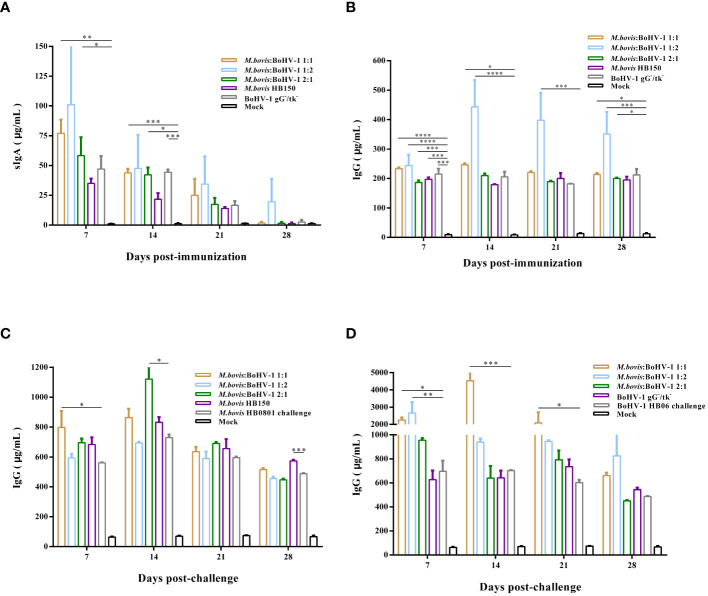
Post- immunization sIgA **(A)** and IgG **(B)** antibody levels were monitored. In addition, we analyzed the Post- challenge IgG antibody levels after *M. bovis* HB0801 **(C)** and BoHV-1 HB06 **(D)** challenge.

Vaccination induced high levels of sIgA antibodies after immunization, with the highest antibody levels induced in the 1:1 and 1:2 groups, whose values reached 77 μg/mL and 101 μg/mL, respectively, indicating that the vaccine can induce mucosal immune response. At days 7 and 14 post immunization, sIgA antibody levels in the 1:1 and 2:1 groups were significantly higher than those of the control (*p* < 0.05) (**Figure 4A**), whereas sIgA antibody levels in all immunized groups did not change after challenge compared to the control group.

IgG plays a key role in the secondary immune response. Here, vaccinated cattle rapidly produced high levels of IgG antibodies after immunization, especially in the 1:2 group, whose levels were significantly higher than those of the control throughout the entire immunization period (*p* < 0.001) (**Figure 4B**). After challenge with *M. bovis* HB0801, the IgG antibody levels in all immunized groups increased, especially in the 1:1 and 2:1 groups. On days 7 and 14 after challenge, the IgG antibody levels of these two groups differed from those of the non-immune challenge group, respectively (*p* < 0.05). IgG antibody levels dramatically increased in the immunized groups challenged by BoHV-1 HB06, with the most pronounced elevation to 4534 μg/mL in the 1:1 group at 14 days post challenge. The IgG level of 1:1 group was significantly higher than non-immune challenge group from day 7 to day 21 after challenge (*p* < 0.05) (**Figures 4C, D**). Overall, the 1:2 group induced the highest levels of sIgA and IgG antibodies after immunization compared with all other immunized groups; however, combined with the results after *M. bovis* HB0801 and BoHV-1 HB06 challenge, the 1:1 group had a more balanced performance.

### 3.5 *M. bovis*-BoHV-1 combined vaccine induce cellular immunity in cattle

**Figure 5:**
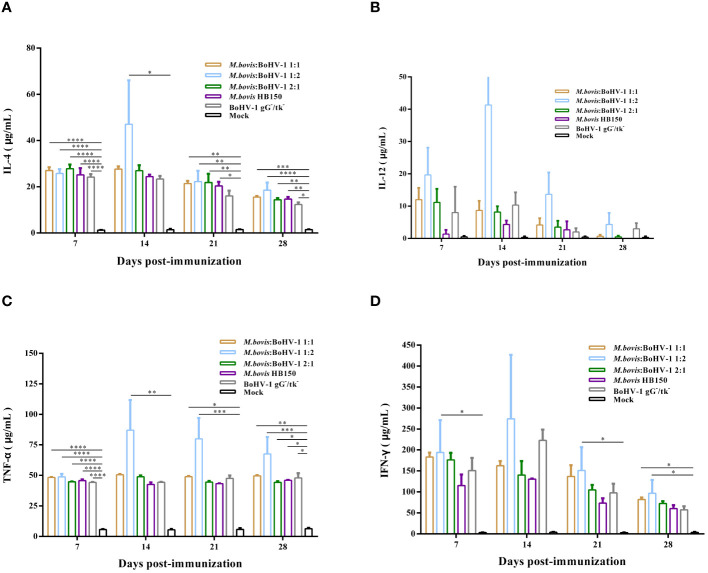
Immune response following immunization of cattle with *M. bovis*-BoHV-1 combined vaccine. **(A–D)** represent the level of IL-4, IL-12, TNF-α and IFN-γ, respectively. At least 3 independent replicates for each experimental group.

After vaccination, IFN-γ levels were significantly higher in all immunized groups, and levels of IL-4, IL-12, and TNF-α slightly increased in all vaccinated groups, but the level of IL-12 produced by all experimental groups was not significantly different from that of the control group. The 1:2 group induced the strongest cellular immune response of all groups. Thus, the attenuated and marker *M. bovis*-BoHV-1 combined vaccine induced a mixed Th1/Th2 response biased toward Th1 in cattle after immunization (**Figures 5A–D**).

**Figure 6:**
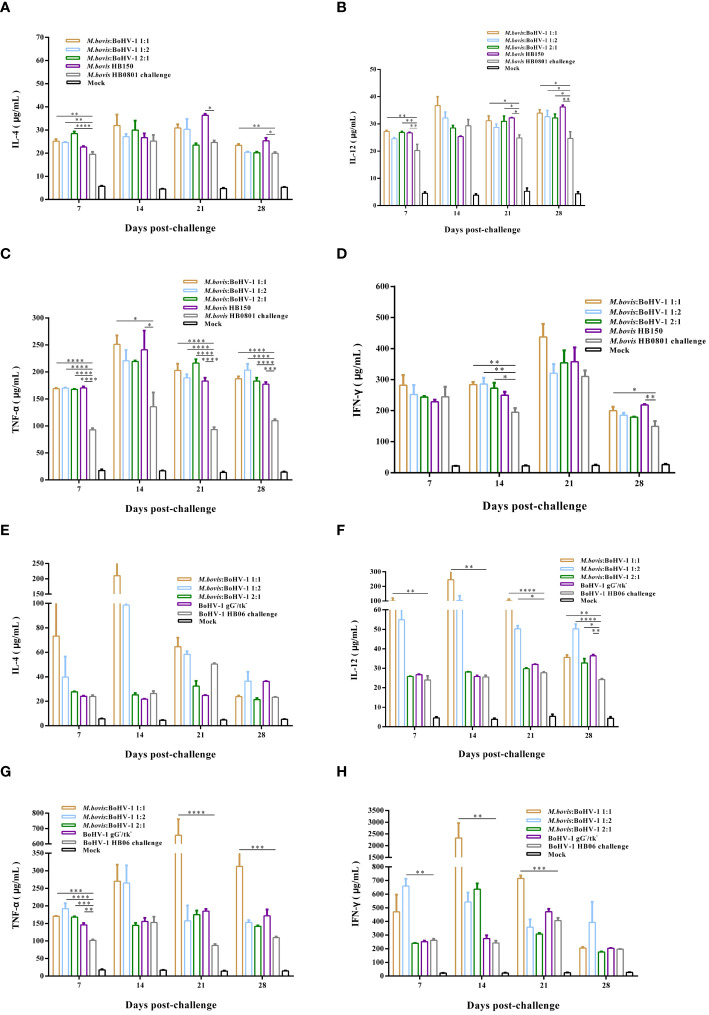
Immune response induced in vaccinated cattle challenged with *M. bovis* HB0801 **(A–D)** and BoHV-1 HB06 **(E–H)**. Each experimental group had at least 3 independent replications to obtain the results of IL-4, IL-12, TNF-α and IFN-γ.

TNF-α, and IFN-γ levels were significantly elevated after challenge with *M. bovis* HB0801, while the concentration of IL-4 and IL-12 experienced small increases. Levels of these cytokines in immunized groups were significantly higher than those in the non-immune but challenged group (*p* < 0.05). The 1:1 group showed levels as high as 250 μg/mL and 400 μg/mL of TNF-α and IFN-γ at 14 and 21 days post challenge, respectively. The vaccine likely induced a mixed Th1/Th2 response with a Th1 bias after challenge with *M. bovis* HB0801 (**Figures 6A–D**).

After challenge with BoHV-1 HB06, levels of IL-4, IL-12, TNF-α, and IFN-γ were significantly higher than those before challenge. The 1:1 group showed the highest levels compared with the non-immune but challenged group (*p* < 0.01), and the IFN-γ level peaked at 2000 μg/mL 14 days post challenge. Thus, the vaccine induced a mixed Th1/Th2 response in cattle challenged with BoHV-1 HB06 (**Figures 6E–H**).

After immunization, the 1:2 group elicited the most intense cellular immune response among all vaccinated groups. Moreover, after *M. bovis* HB0801 and BoHV-1 HB06 challenge, the 1:1 group generated much stronger cellular immunity against both pathogens simultaneously.

### 3.6 *M. bovis*-BoHV-1 combined vaccine-mediated immune cell activation in the lung and spleen tissues

The lung is one of the most important target organs for *M. bovis* and BoHV-1 infections, and the spleen is an important lymphoid organ. Therefore, lung and spleen tissues of vaccinated and unvaccinated cattle were analyzed to measure the proliferation of immune cells. In which, CD4^+^ and CD8^+^ target T cells, CD19^+^ targets B cells, CD11b^+^ targets macrophages cells and CD11c^+^ targets dendritic cells. As shown, macrophages and dendritic cells in the lungs and spleen significantly increased in the immunized group represented by 1:1 after challenge with *M. bovis* HB0801 or BoHV-1 HB06, and their levels were significantly different from those in the non-immune challenge group (*p* < 0.01) (**Supplementary Figure 3**). Moreover, the number of CD4^+^, CD8^+^, and CD19^+^ cells in the lung and spleen of the immunized groups increased and statistically differed from the non-immune challenge group after being challenged with *M. bovis* HB0801 or BoHV-1 HB06 (*p* < 0.05) (**Supplementary Figure 3**).

These results indicate that the vaccinated cattle were better able to activate macrophages and dendritic cells, which delivered and phagocytosed antigens to elicit an immune response through the humoral circulation. Additionally, the high abundance of CD4^+^, CD8^+^, CD19^+^ cells detected in vaccinated cattle indicated that the *M. bovis*-BoHV-1 combined vaccine significantly upregulated cellular and humoral immune responses and promoted Th1/Th2 balance in cattle, especially the 1:1 group. The vaccine may maintain and reactivate the cellular and humoral immune response in cattle after challenge.

### 3.7 Evaluation of gross lesion and micropathological injury

In the immunized groups, lung damage was less severe and was concentrated mainly in the lobule of the lungs, with a mild degree of bruising and fleshy lesions. Lesions in the unimmunized but challenged groups were more severe and throughout the lungs, with extensive bruising and carnification throughout the lungs of cattle challenged with *M. bovis* HB0801; cattle challenged with BoHV-1 HV06 had hemorrhages in the lobes with interlobular adhesions and granulomatous changes (**Supplementary Figure 4**).

No significant pathological changes were found in any of the immunized groups after *M. bovis* HB0801 or BoHV-1 HB06 challenge; the alveolar structure was relatively complete with no inflammatory exudate in the alveolar lumen. Only the 1:2 group had a small amount of inflammatory cell exudation in the alveolar cavity after *M. bovis* HB0801 challenge. There were varying degrees of alveolar wall hyperplasia but little disruption of the structure; only the alveoli in the 2:1 group fused into larger alveolar cavities after BoHV-1 HB06 challenge. In contrast, unvaccinated calves challenged with *M. bovis* HB0801 presented the most severe lesions and loss of normal lung tissue structure, interstitial hyperplasia, and inflammatory infiltrates (**Supplementary Figure 5**). Alveolar morphology of BoHV-1-challenged cattle showed some interstitial hyperplasia with inflammatory cell exudation (**Supplementary Figure 6**).

The morphology of the lung tissue of immunized cattle was more complete than that of unimmunized cattle following challenge with *M. bovis* HB0801 and BoHV-1 HB06. Ultimately the vaccine showed the most balanced efficacy, particularly in the 1:1 group, providing sufficient protection.

## 4 Discussion

Interactions between the host and the respiratory microbiota can maintain cattle health to some extent and resist colonization by pathogenic microorganisms (17, 18). However, some pathogenic microorganisms appear to be conditionally pathogenic, especially in newly weaned calves, whose many stressors can disrupt the respiratory system and promote development of BRD (19, 20). Pathogenic microorganisms associated with common BRD pathogens such as *M. bovis*, *Mh*, and *Pm* have been observed in nasal and pharyngeal swabs from both healthy and BRD-infected cattle (1, 21). Common respiratory pathogens have also been recently identified in the lungs of both healthy and affected cattle. Current evidence suggests that although *M. bovis* is a minor pathogen of the bovine respiratory tract (22), its co-occurrence with other pathogenic factors must be considered. Mutual cooperation between respiratory bacteria and viruses has been reported in humans (23); thus, we hypothesize that the coexistence of *M.bovis* with bovine respiratory viruses such as BoHV-1 also leads to changes in the respiratory microecological environment. Transient immunosuppression induced by BoHV-1 renders cattle more susceptible to secondary bacterial infections including *M. bovis* and *Mh*, leading to BRD. The virulence of pathogenic microorganisms in the host also increases under these conditions.

Vaccination is the most direct and effective way to prevent and control BRDC in feedlots, but vaccine development remains challenging because BRDC is multipathogenic; therefore, combined and multivalent vaccines must be developed to combat different pathogens. In Europe, there are some monovalent or multivalent vaccines that can be used to prevent BRDC (24, 25). Modified live virus (MLV) vaccines are known to induce effective humoral and cellular immune responses. In contrast, inactivated vaccines can elicit a strong humoral immune response but require multiple inoculations to achieve this (26, 27). Therefore, MLV vaccines are more effective than inactivated vaccines to prevent and control BRDC, but the MLV vaccines still carry the risk of shedding the virus. However, there is currently no combined live and marker vaccine.

To combat these challenges, we developed a novel attenuated and marker *M. bovis*-BoHV-1 combined vaccine and evaluated its safety based on clinical signs after vaccination. Rectal temperatures of the cattle did not increase for several days after vaccination, only transiently rising, which is normal post vaccination. The cattle also showed no obvious respiratory signs after vaccination. Subsequently, we assessed the protective efficacy of the attenuated *M. bovis*-BoHV-1 combined vaccine. As expected, the combined vaccine induced a strong humoral and cellular immune response both after immunization and after challenge with *M. bovis* HB0801 or BoHV-1 HB06. This is a pleasing result because both humoral and cellular immune responses are required for protection against BoHV-1 (28). The vaccine-induced antibody response is one of the most important immunological factors against infections. Correspondingly, when those vaccinated cattle were challenged, rapidly produced high titers of antibodies against various pathogens simultaneously, indicating that the *M. bovis*-BoHV-1 vaccine induced a specific humoral immune response in cattle. However, protection against secondary or recurrent infection is mainly attributed to the cell-mediated immune response (CMI) rather than the humoral immune response (29), where CD4^+^ T cells regulate both cellular and humoral immune responses, and CD8^+^ T cells (cytotoxic T lymphocytes) kill infected cells and inhibit the spread of intracellular pathogens (30). In CMI, Th1 cells are primarily combat intracellular pathogens and promote the production of cytokines such as IFN-γ, which is small molecule protein that regulate the immune system through potent antiviral activity and promotion of other immune effector functions (31). Among these, the level of IFN-γ secretion is positively correlated with protective effects and reduced clinical signs in infected animals (32). The cattle in this study accordingly produced high levels of IFN-γ after vaccination and *M. bovis* HB0801 or BoHV-1 HB06 challenge. In addition, pro-inflammatory cytokines such as IFN-γ and TNF-α can increase the expression of IL-12 and regulate the biological function of IL-12, whose levels were higher in the immunized groups after challenge. It is unclear which T cell subsets release IFN-γ during infection with these two pathogens, our future work should address these issues. The positivity rates of CD4^+^ T cells, CD8^+^ T cells, and CD19^+^ cells in the lungs and spleens of immunized cattle were higher than those of the control group or challenged group. These results also suggest that the *M. bovis*-BoHV-1 combined vaccine enhanced the immune function of T and B lymphocytes in peripheral blood and target organs.

Antibodies are produced during the Th2 response (33). sIgA is the most important antibody for local mucosal immunity, determining the resistance of the respiratory mucosa to pathogens, whereas systemic humoral immunity depends on IgG, which plays an important role in anti-infective immunity. The measurement of sIgA and IgG could help to evaluate the vaccine-induced systemic immune response (34). This is consistent with our findings, as vaccinated calves produced high levels of sIgA and therefore a favorable mucosal immune response. However, when experimental groups challenged with *M. bovis* HB0801 or BoHV-1 HB06, the serum IgA level did not elevate, it is suspected that the reduction of serum IgA levels 28 days after immunization may be the cause. Also, the pathogen’s effects on the immune system may prevent the production of sIgA after either the *M. bovis* or BoHV-1 challenge. The specific mechanism requires further exploration. The experimental groups also produced extremely high levels of IgG antibodies after challenge with *M. bovis* HB0801 or BoHV-1 HB06, where the 1:1 group performed best. Other studies have shown that experimental BoHV-1 vaccines can induce early adaptive immune responses characterized by Th1 and Th2 responses (35), and our attenuated *M. bovis*-BoHV-1 combined vaccine could induce high levels of IFN-γ and stimulate the production of IL-4, IL-12 and TNF-α, providing that the combined vaccine can induced Th1-biased mixed Th1/Th2 responses. In general, *M. bovis* is primarily an extracellular pathogen that induces a Th2-type immune response (36); however, it is also associated with a biased differentiation of Th1 cell subsets according to our results. *M. bovis* antigens can enter and survive in host cells. Thus, vaccines that induce both Th1 and Th2 immune responses will likely better prevent and control disease. The two *M. bovis* vaccines Mycomune^®^R and Pulmo-Guard™MpB currently commercially available in the United States did not show significant differences between experimental and control groups in the assessment of selected cytokine responses, but the concentrations of the proinflammatory cytokines IL-1β and TNF-α increased significantly after vaccination (37). In contrast, our results showed that cattle challenged with *M. bovis* HB0801 can produce high levels of TNF-α and IFN-γ and promote the expression of IL-4 and IL-12. This Th1-biased Th1/Th2 mixed response can effectively resist the challenge of *M. bovis*. Moreover, IL-4 plays an important role in inducing and maintaining the Th2 immune response, which ultimately promotes antibody production (38). In summary, vaccination is the most effective tool to reduce the spread of disease and outbreaks, both humoral and cellular immune responses are necessary to control infections, and strong T-cell memory is essential for long-term immunity (28).

We previously evaluated the safety and protective efficacy of the attenuated and marker *M. bovis*-BoHV-1 combined vaccine in a rabbit model, but the levels of humoral and cellular immunity induced by the combined vaccine after immunization were not very strong. However, the combined vaccine induced high levels of humoral and cellular immune responses in cattle, which were consistent with the rabbit experiment, and also proved the real effectiveness of the combined vaccine in this study.

In conclusion, the *M. bovis*-BoHV-1 combined vaccine is promising for the cattle industry. Vaccinated cattle showed no clinical signs and resisted *M. bovis* or BoHV-1 challenge as shown by changes in gross lung damage in unvaccinated but not vaccinated cattle. In addition, the functions of T and B lymphocytes in target organs and immune organs of immunized cattle significantly improved compared to those of the control and unimmunized ones. The protective effect was best at a 1:1 ratio of the two antigens all immunized groups when challenged with *M. bovis* HB0801 or BoHV-1 HB06. However, the optimal immunization dose and safety of the vaccine warrant further studies to maximize its efficacy, stability, and safety in calves at different ages and growth stages.

## Data availability statement

The original contributions presented in the study are included in the article/**Supplementary Material**. Further inquiries can be directed to the corresponding authors.

## Ethics statement

The animal study was approved by the Animal Experiment Ethics Committee of Huazhong Agricultural University and conducted in strict accordance with the Guidelines for the Care and Use of Laboratory Animals of Wuhan, Hubei, China (Huazhong Agricultural University Ethics Approval Number: HZAUCA-2023-0038). The study was conducted in accordance with the local legislation and institutional requirements.

## Author contributions

SZ: Conceptualization, Formal analysis, Investigation, Methodology, Resources, Software, Visualization, Writing – original draft. GL: Formal analysis, Investigation, Methodology, Resources, Writing – original draft. YSZ: Formal analysis, Investigation, Methodology, Resources, Validation, Writing – original draft. CW: Investigation, Methodology, Validation, Writing – original draft. XX: Investigation, Methodology, Validation, Writing – original draft. YHZ: Investigation, Methodology, Validation, Writing – original draft. ZX: Investigation, Methodology, Validation, Writing – original draft. WW: Investigation, Methodology, Validation, Writing – original draft. LY: Conceptualization, Formal analysis, Project administration, Writing – original draft. JC: Conceptualization, Project administration, Writing – original draft. AG: Conceptualization, Funding acquisition, Project administration, Supervision, Writing – review & editing. YC: Conceptualization, Funding acquisition, Project administration, Supervision, Writing – review & editing.
