# Supplementary material for: Investigation of the safety and protective efficacy of an attenuated and marker M. bovis-BoHV-1 combined vaccine in bovines
Source: Front Immunol. 2024 Apr 4;15:1367253. doi: 10.3389/fimmu.2024.1367253 (PMC11027501; doi:10.3389/fimmu.2024.1367253)
Supplement: Supplementary file 1 [file Presentation_1.pdf]

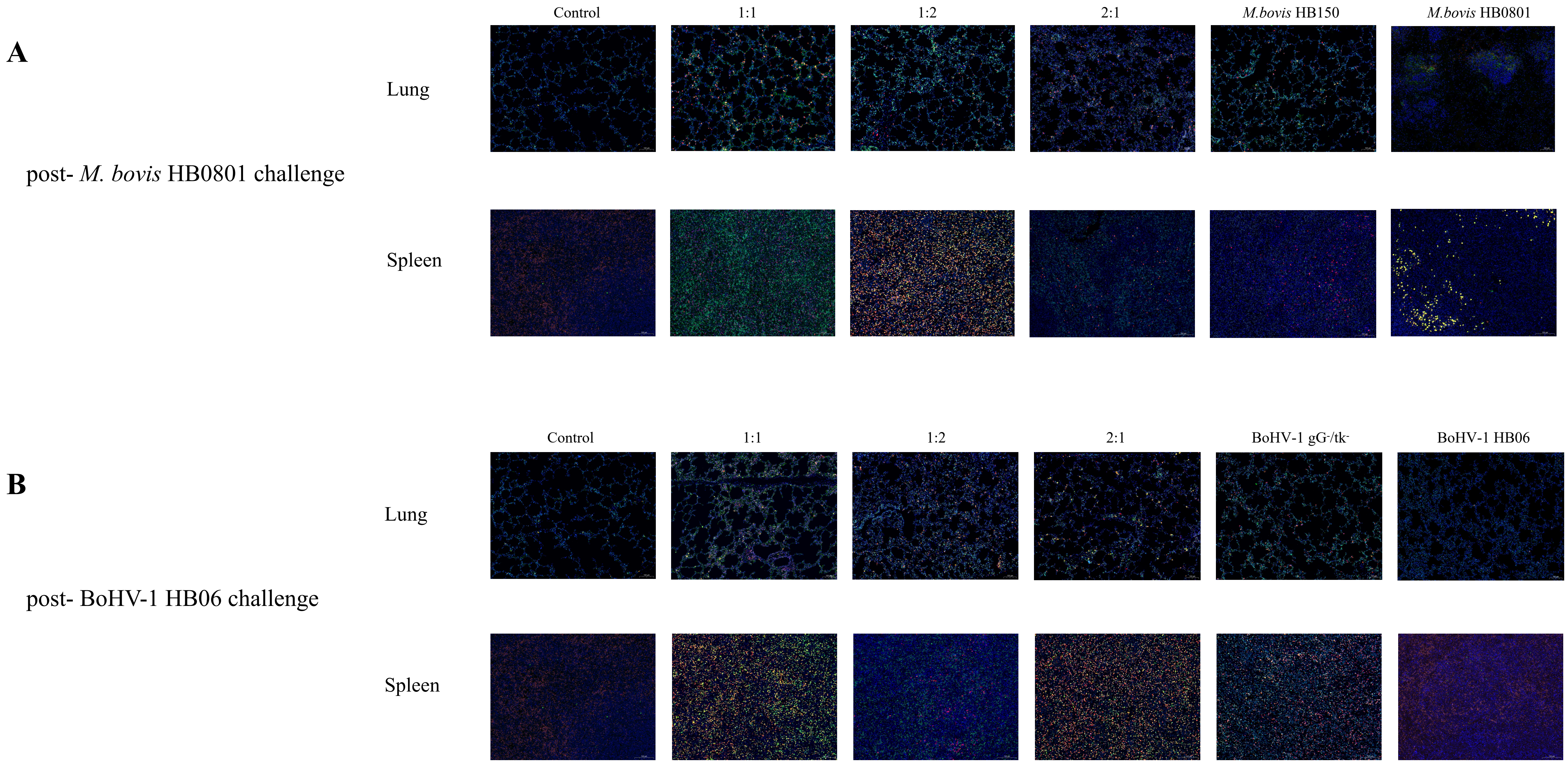

Figure S1. Cellular distribution of lung and spleen tissues in cattle after **(A)** *M. bovis* HB0801 and **(B)** BoHV-1 HB06 challenge. Green fluorescence represents CD11C^{+} cells and red fluorescence represents CD11B^{+} cells.

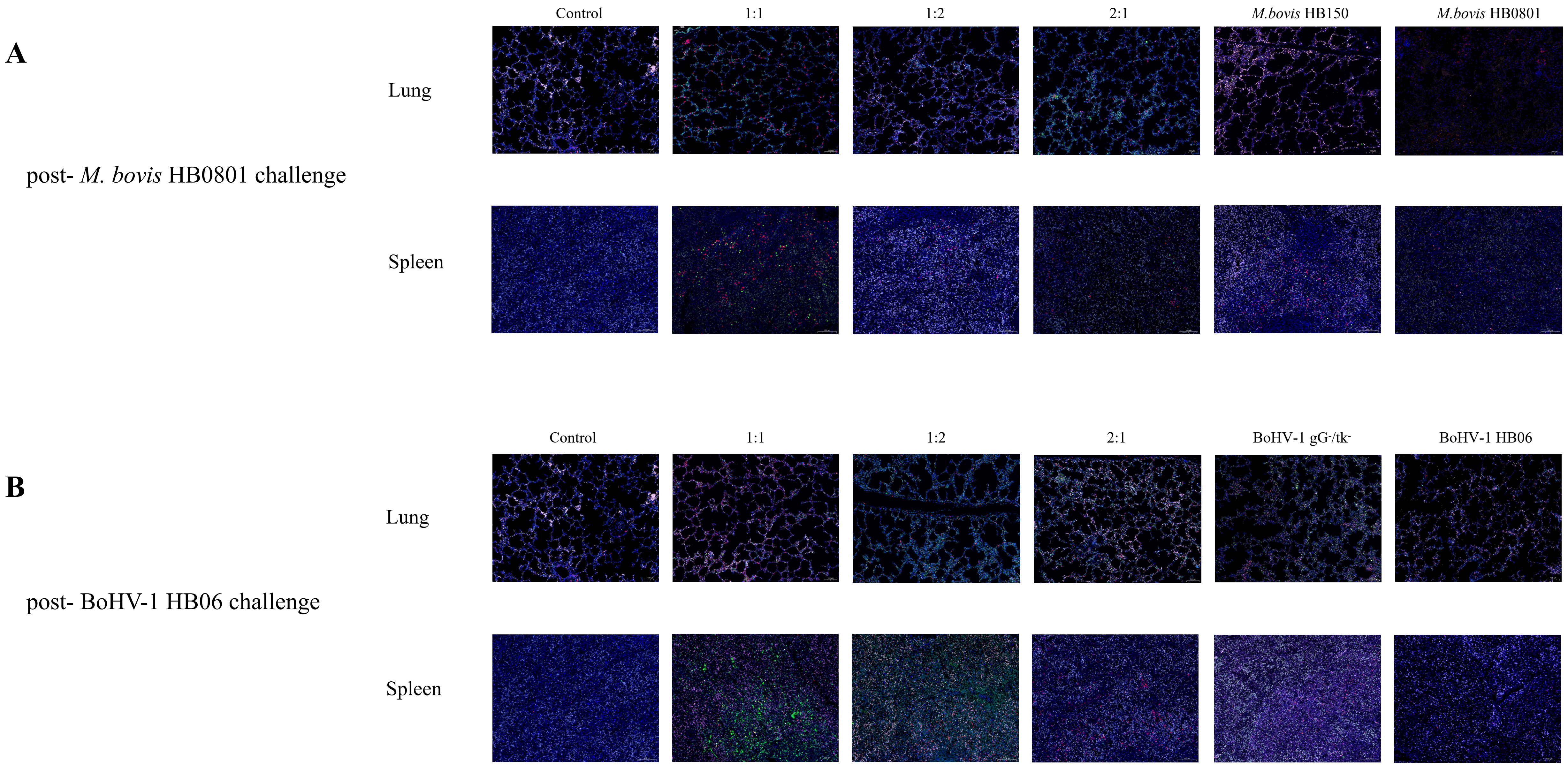

Figure S2. Cellular distribution of lung and spleen tissues in cattle after **(A)** *M. bovis* HB0801 and **(B)** BoHV-1 HB06 challenge. Green fluorescence represents CD19$^+$ cells, red fluorescence represents CD4$^+$ cells and pink fluorescence represents CD8$^+$ cells.

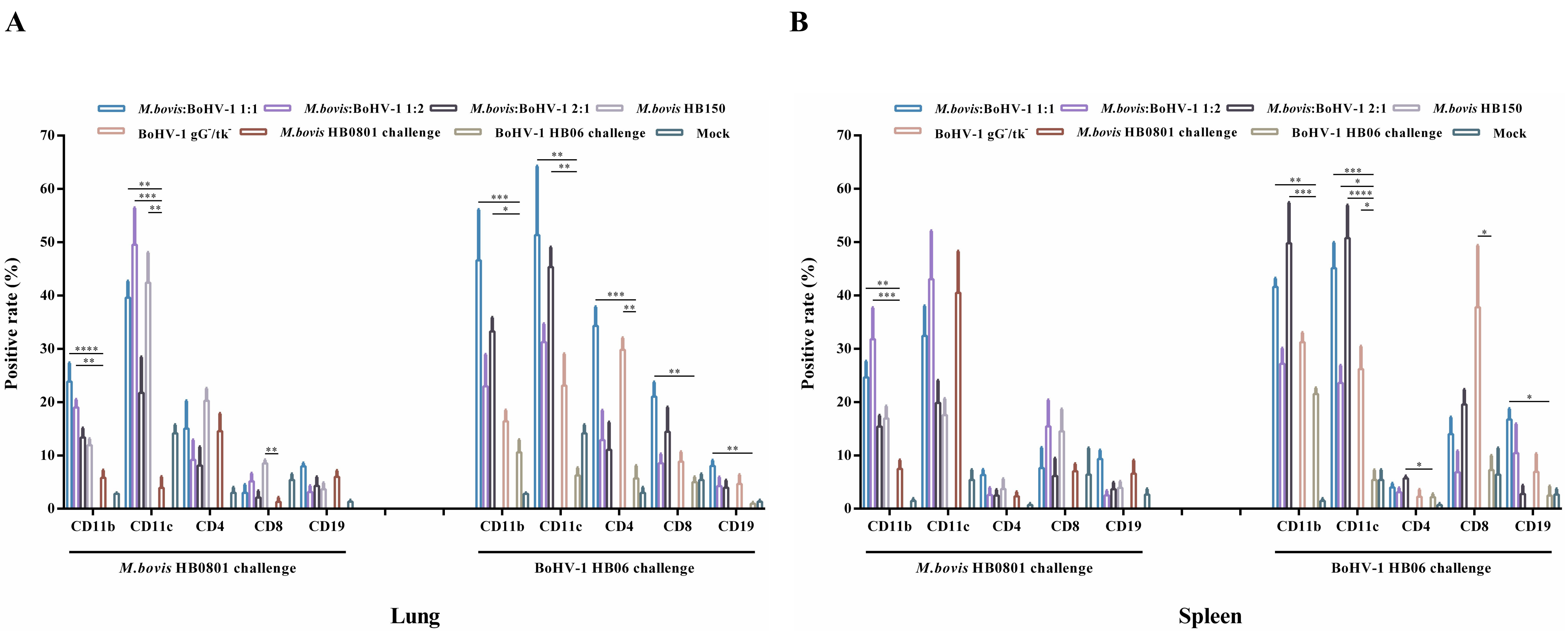

Figure S3. After *M. bovis* HB0801 and BoHV-1 HB06 challenge, immunofluorescence analysis of different types of cells in the lung **(A)** and spleen **(B)** tissues were performed. The positive rate means the ratio of the number of positive cells to the total number of cells.

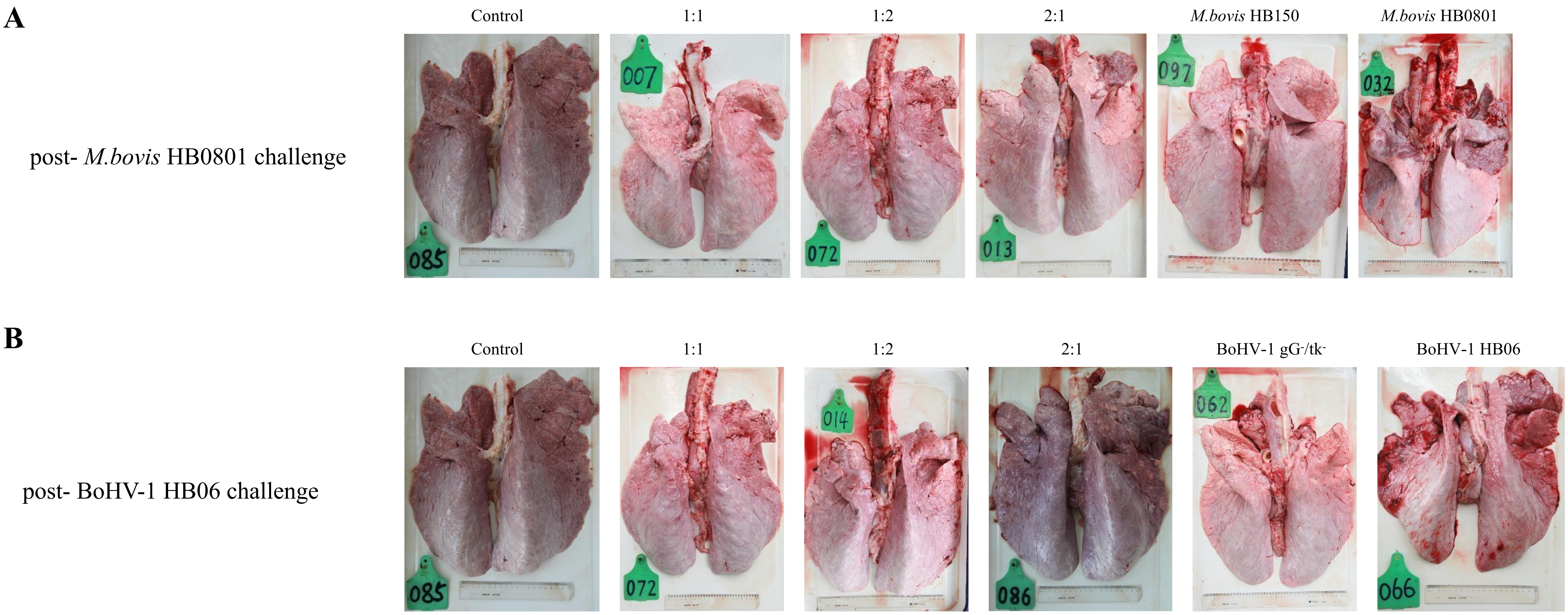

Figure S4. Gross lesions of lung tissue after *M. bovis* HB0801 **(A)** or BoHV-1 HB06 **(B)** challenge.

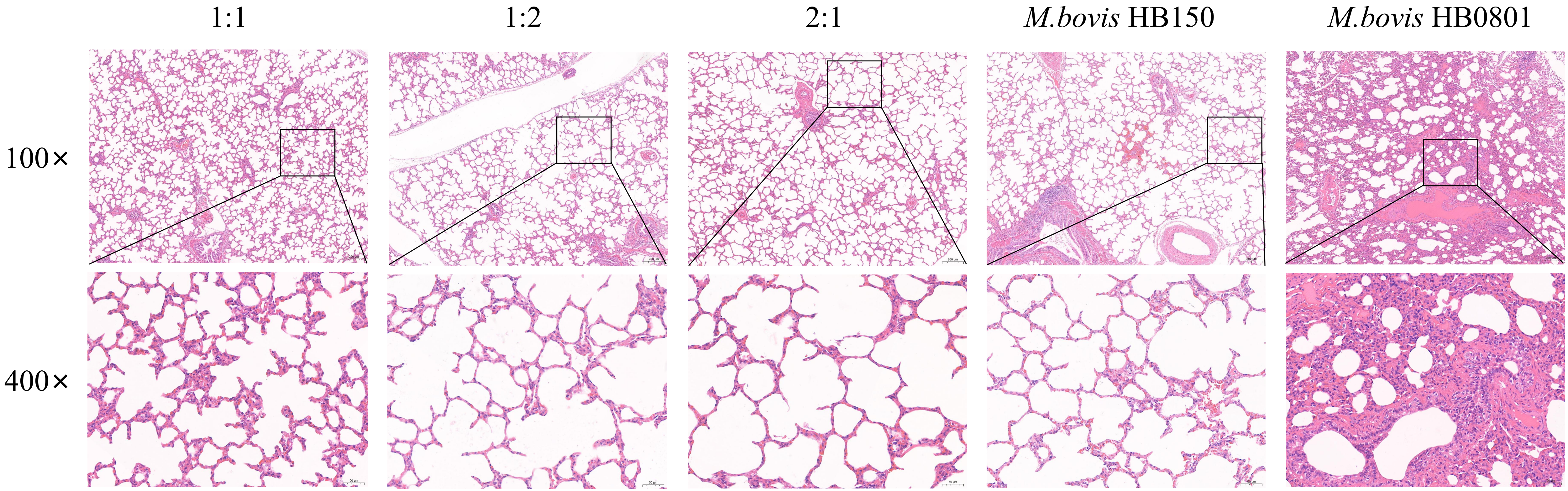

Figure S5. Histopathological images of lung tissues after *M. bovis* HB0801 challenge tained by H&E. The scale sizes are 200μm (top) and 50μm (bottom), respectively. The figure below is an enlargement of part of the area of the upper figure.

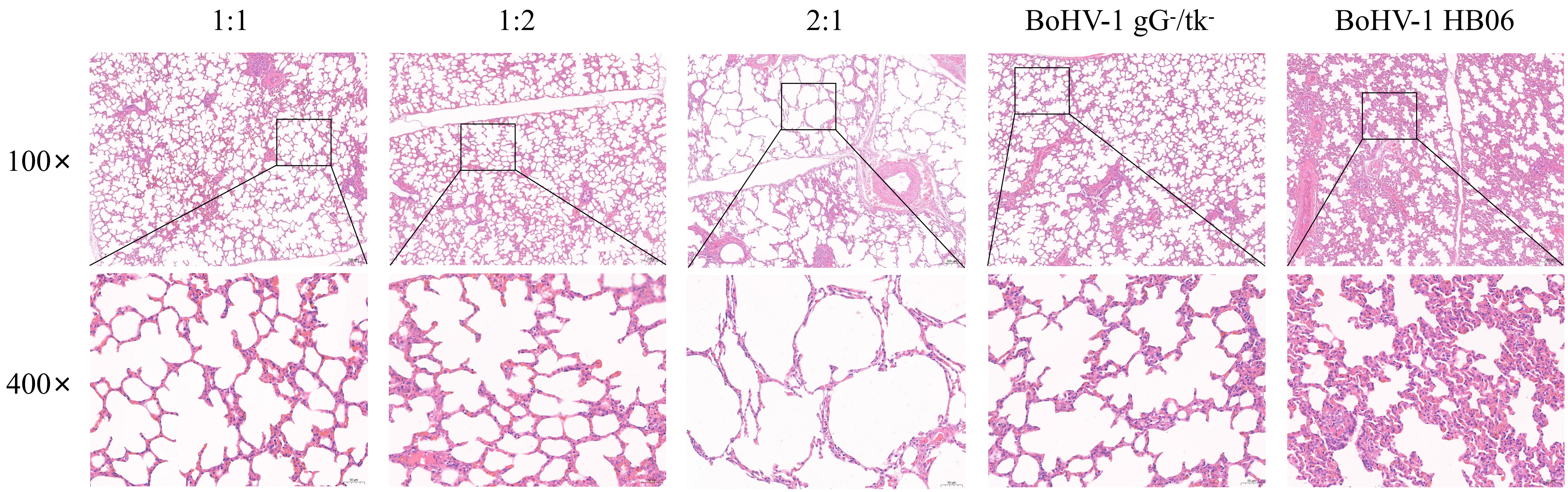

Figure S6. Histopathological images of lung tissues after BoHV-1 HB06 challenge tained by H&E. The scale sizes are 200μm (top) and 50μm (bottom), respectively. The figure below is an enlargement of part of the area of the upper figure.
